# Surgical Treatment of Enlarged Cervical Leiomyoma with Concomitant Uterine Prolapse: A Case Report

**DOI:** 10.3390/jcm13144210

**Published:** 2024-07-19

**Authors:** Ah-Yun Song, Ju-Young Bae, Jin-Sol Park, Tae-Hyun Kim

**Affiliations:** 1 Department of Obstetrics and Gynecology, Konyang University Hospital, Department of Medicine, Konyang University College of Medicine, Daejeon 35365, Republic of Korea; ky400874@kyuh.ac.kr; 2 Department of Obstetrics and Gynecology, Konyang University Hospital, Daejeon 35365, Republic of Korea; 400954@kyuh.ac.kr; 3 Department of Obstetrics and Gynecology, Seoul National University Hospital, Seoul 13520, Republic of Korea; wlsthf08321@gmail.com

**Keywords:** pelvic organ prolapse, cervical leiomyoma, sacrocolpopexy, Da Vinci Xi

## Abstract

This case report details the surgical treatment of a rare enlarged cervical leiomyoma with uterine prolapse in a 48-year-old woman. She presented to Konyang University Hospital with a palpable vaginal mass, lower abdominal pain, and urinary incontinence. Despite being nulliparous, she had severe chronic constipation due to schizophrenia medication and lived in a health care facility separated from her family. Pelvic examination revealed stage 3 uterine prolapse with a large necrotic cervical leiomyoma. A robot-assisted vaginal hysterectomy followed by sacrocolpopexy was performed using the Da Vinci Xi Surgical System. Histopathology confirmed cervical leiomyoma with squamous metaplasia. At a three-month follow-up, there were no complications, pelvic anatomy was restored, and urinary incontinence improved. Although the patient had a systemic infection due to the necrotic cervical leiomyoma, raising concerns about the increased risk of infection associated with mesh use, she was high-risk for pelvic organ prolapse (POP) recurrence due to her medical history and living situation. Therefore, she underwent concurrent surgeries with pre- and postoperative antibiotic treatment, and recovered without complications. Given that the risk of developing POP increases after a hysterectomy, in high-risk patients, as demonstrated in this case, the concurrent surgical correction of POP may be an effective strategy.

## 1. Introduction

Uterine leiomyoma is the most common benign tumor of the uterus, affecting approximately 40–50% of women over the age of 35 [1]. However, cervical leiomyoma is relatively rare, with a prevalence of less than 5% [2]. While cervical leiomyomas are often asymptomatic, they can present with symptoms such as vaginal bleeding, lower abdominal pain, urinary frequency, dyspareunia, and constipation. Complications, although infrequent, may include prolapse, thromboembolism, acute torsion, urinary retention, and renal failure [3]. Pelvic organ prolapse (POP) occurs when organs such as the bladder and uterus descend or protrude through the vaginal wall from their normal positions due to weakened or damaged supporting structures of the pelvic floor [4]. POP may be asymptomatic, but common symptoms include vaginal or pelvic pressure, a sensation of vaginal bulge, or the feeling of something protruding from the vagina [5]. Additionally, POP is often associated with urinary storage and voiding disorders, including urinary retention, urgency, and incontinence [6]. Cervical leiomyomas, in particular, can exacerbate POP. The weight of these benign tumors, especially submucosal or pedunculated leiomyomas, can pull the uterus downward along with the bladder and vaginal wall, resulting in cervical elongation [7,8]. This case report highlights a rare instance where an enlarged cervical leiomyoma was associated with significant uterine prolapse. The condition was effectively managed through a robotic-assisted vaginal hysterectomy followed by sacrocolpopexy (SCP), illustrating an effective surgical approach for such complex presentations.

## 2. Case Report

A 48-year-old woman with a medical history of hypertension, hypothyroidism, and schizophrenia presented to the outpatient department of Obstetrics and Gynecology at Konyang University Hospital, complaining of a palpable mass in her vagina. The patient also reported lower abdominal pain and intermittent urinary incontinence. She was unmarried, nulliparous, and had regular menstruation. Her height was 151 cm, weight 63 kg, and BMI 27.63. She resided in a healthcare facility and had been regularly taking laxatives due to severe chronic constipation.

**Figure 1:**
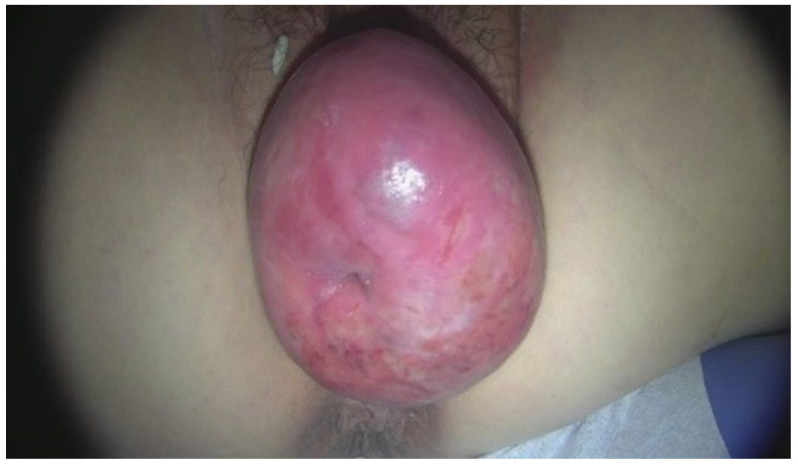
On physical examination, the cervix is protruding through the vaginal opening, and there is observed necrosis of the cervix.

The patient has been experiencing lower abdominal discomfort since 2003. In 2010, she first noticed a bulging mass in her vagina that she was able to manually reduce, but recently the mass had increased in size and manual reduction was no longer effective. Pelvic examination revealed that the cervix was enlarged to the size of a woman’s fist and exhibited necrotic, ulcerative lesions (Figure 1). The diagnosis of cervical leiomyoma and stage 3 uterine prolapse was made, and a robot-assisted SCP was planned. Given the patient’s intermittent stress incontinence symptoms, a consultation with the urology department was conducted to discuss the need for intervention to prevent the risk of de novo stress urinary incontinence following SCP. It was decided to follow up on her symptoms postoperatively to determine if there was an improvement after the robot-assisted SCP.

The patient was admitted earlier than the scheduled surgery date due to severe pain and a high fever (38 °C). Ultrasound and abdominal pelvic computed tomography (APCT) revealed vaginal and bladder prolapse and mild paralytic ileus of the bowel. Empirical antibiotic therapy with ceftriaxone and metronidazole was initiated, considering the potential for infection in the prolapse area. Blood, urine, and sputum cultures were negative for specific pathogens; however, Enterococcus faecalis was detected in the vaginal culture. Consequently, the antibiotic regimen was adjusted to ciprofloxacin based on the antibiotic susceptibility results. After an 8-day course of antibiotic therapy and pain management, the scheduled surgery was performed on the planned date. The surgery was conducted using the Da Vinci Xi Surgical System (Intuitive Surgical, Inc., Sunnyvale, CA, USA), and the surgical procedures were as follows.

**Figure 2:**
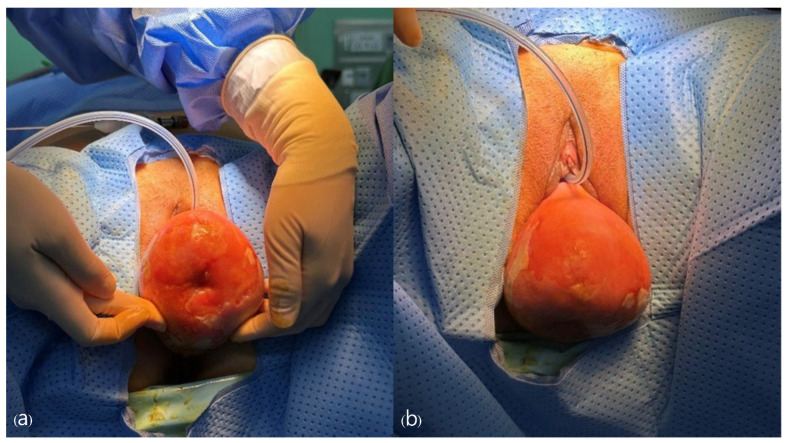
Appearance confirming uterine prolapse prior to surgery, observed (**a**) from the front, (**b**) from above.

Under general anesthesia, the patient was placed in the dorsal lithotomy position and draped appropriately. A pelvic examination was performed, and a Foley catheter was inserted into the bladder (Figure 2). An 8 mm umbilical incision to the fascia was made, and a Veress needle was inserted to establish pneumoperitoneum with carbon dioxide gas inflation to 12 mmHg. We used one 8 mm camera port placed 2 cm above the umbilicus. An 8-mm robotic port was placed in the left upper quadrant, and two 8 mm robotic ports were placed in the right upper quadrant, each positioned at least 8 cm apart laterally. A 12 mm assistant laparoscopic port was placed in the left upper quadrant, between the robotic port and the camera port. A 15° Trendelenburg position was obtained to ensure visibility of the entire pelvis. The robot was positioned on the right side of the patient and docked. Robotic EndoWrist instruments (Intuitive Surgical, Sunnyvale, Inc., CA, USA) were utilized, including monopolar curved scissors, fenestrated bipolar forceps, and the mega-needle driver.

**Figure 3:**
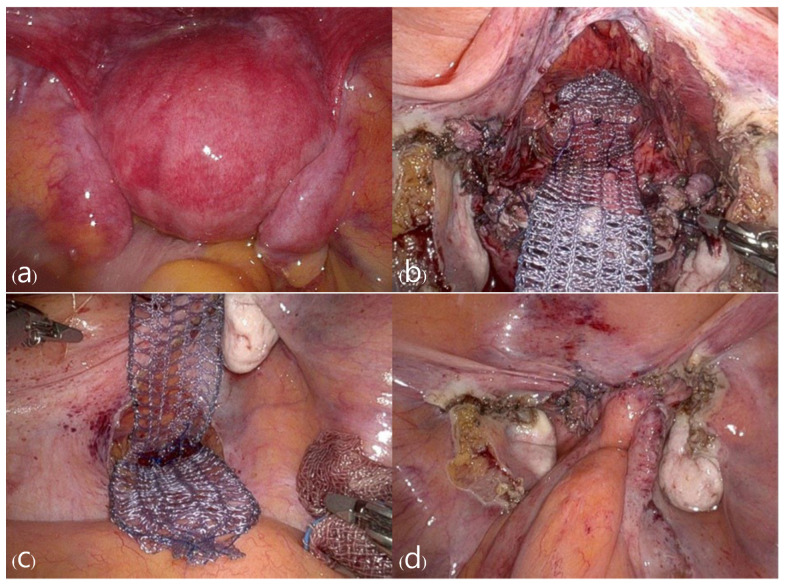
Pictures of the surgical procedure: (**a**) The uterine prolapse within the abdominal cavity before surgery, (**b**) appearance after hysterectomy with mesh fixation to the stump, (**c**) fixation of mesh to the sacral promontory, (**d**) mesh was covered by the peritoneum in the end.

**Figure 4:**
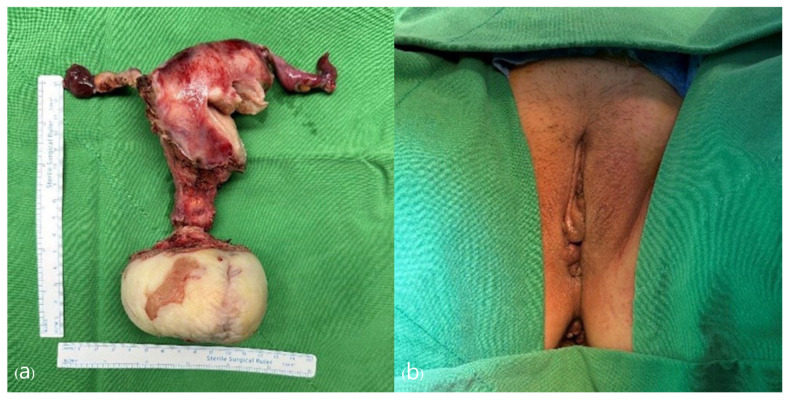
(**a**) Cervical leiomyoma with squamous metaplasia. (Total size: 13.0 × 10.0 × 6.5 cm). (**b**) Post-operation appearance. The uterine prolapse has completely recovered.

Upon entering the abdominal cavity, the prolapsed uterus was observed. Both round ligaments were electrocoagulated and divided, and the retroperitoneal space was developed. Both fallopian tubes and utero-ovarian ligaments were electrocoagulated and divided, preserving both ovaries. Due to the severe utero-vaginal prolapse, the remaining steps of the hysterectomy were performed via a vaginal approach. The uterus was pulled down into the operative field by applying traction on each lateral side of the cervical lip. After developing the vesicovaginal space, anterior and posterior colpotomy were performed. Both uterosacral and cardinal ligaments were clamped with a Heaney clamp, cut, and suture-ligated using 2-0 Polysorb (Medtronic, Minneapolis, MN, USA). After the uterus was removed, the remainder of the surgical procedure was completed using the Da Vinci Xi Surgical System. The vaginal vault was continuously sutured using 2-0 Monofix (Samyang Biopharm, Seoul, Republic of Korea). The vesicovaginal and rectovaginal spaces were dissected. A partially absorbable Y-type mesh (Seratex, Serag-Wiessner, Naila, Germany) was sutured to the anterior and posterior vaginal walls using 2-0 PDS (Ethicon, Somerville, NJ, USA) with at least 10 interrupted sutures. The posterior abdominal peritoneum was opened through the sacral promontory. The other side of the mesh was anchored to the anterior longitudinal ligament at the level of the S1 vertebral body using two stitches of 2-0 Prolene (Ethicon, Somerville, NJ, USA). The peritoneum was completely closed using 3-0 Stratafix (Ethicon, Somerville, NJ, USA) (Figure 3 and Figure 4). The total operation time was 300 min, and the estimated blood loss was approximately 100 mL.

Following the surgery, the patient received a combination of Ertapenem and Metronidazole for a total of 14 days due to an extended-spectrum beta-lactamase (ESBL)-positive bacterial infection identified in the pre-operative vaginal culture. The infection was successfully eradicated, and the patient was subsequently discharged.

**Figure 5:**
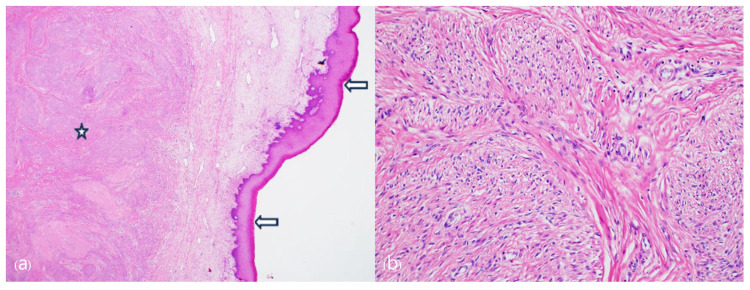
Microscopically leiomyoma lesion is present in the exocervical stromal area (asterisk). Overlying the tumor, exocervical mucosa shows marked hyperkeratosis (arrows) indicating uterine prolapse. (**a**) Low scan magnification, (**b**) high magnification of the leiomyoma lesion showing lobular proliferation of the neoplastic spindle cells (Hematoxylin and Eosin, Original magnification, (**a**) ×20, (**b**) ×100).

The final histopathological examination revealed a cervical leiomyoma with squamous metaplasia, measuring 13.0 × 10.0 × 6.5 cm. Microscopically, hyperkeratosis related to uterine prolapse was noted (Figure 5). At a follow-up observation conducted 3 months postoperatively, no complications were observed, and the pelvic anatomy had returned to normal. Additionally, the patient showed improvement in urinary incontinence symptoms, as confirmed by a urology examination.

## 3. Discussion

This case report details the surgical treatment of a rare case of enlarged cervical leiomyoma with concomitant uterine prolapse. Notably, the patient had not experienced childbirth, typically a major risk factor for uterine prolapse [9]. Instead, the patient’s high BMI and chronic constipation, combined with continuous downward traction from the cervical leiomyoma, likely contributed to the development of the uterine prolapse. The patient presented with symptoms including a sensation of heaviness in the lower abdomen and a protruding vaginal mass over an extended period. However, the patient had a psychiatric history, impairing her awareness of the medical condition and ability to report symptoms. Additionally, she resided in a healthcare facility, separated from her family, and did not receive appropriate treatment until her symptoms worsened. At her initial outpatient visit, necrosis and ulcerative lesions were observed on the cervix, which had been exposed to the external environment for an extended period, indicating an infection requiring long-term antibiotic treatment.

SCP is considered an excellent choice for the treatment of POP due to its low recurrence and reoperation rates compared to other surgical methods [10,11,12]. Most surgical treatments for cervical leiomyoma typically involve myomectomy or hysterectomy, even in cases where POP is present, without concurrent correction of the prolapse [5,13,14]. A study of 247 women who underwent hysterectomy for various benign indications and were followed up 16 years post-surgery found that 153 women (62%) experienced stage 2 or higher POP [15]. Among these, 80 women (52%) reported moderate to severe pelvic floor symptoms, with significantly more long-term pelvic floor symptoms in those with pre-existing POP before hysterectomy compared to others (57% vs. 40%, *p* = 0.009) [15]. In previous cases of patients with cervical myoma, with or without POP, where only hysterectomy was performed, there is no long-term follow-up data available to confirm the outcomes [5,13,14]. However, it is reasonable to assume that the risk of POP recurrence or the re-emergence of pelvic floor symptoms remains high. In this case, the patient already had a high fever due to an infection from the necrotic cervical leiomyoma lesion, raising concerns about the infection risk associated with performing SCP using mesh. However, the patient was overweight and suffered from chronic constipation due to psychiatric medications, making her a high-risk candidate for the recurrence of POP if only myomectomy or hysterectomy were performed. Additionally, due to the difficulty of regular outpatient visits, timely diagnosis and additional surgery for recurrent POP would be challenging. Therefore, despite the increased risk of infection, it was determined that performing SCP in conjunction with a hysterectomy, rather than conducting a hysterectomy alone, was the better option. Consequently, the decision was made to proceed with the concomitant surgeries while administering antibiotic treatment both pre- and post-operatively.

Hysterectomy with SCP can be performed via open or minimally invasive surgery. Due to its superiority in postoperative morbidity, patient recovery, and length of hospital stay, minimally invasive surgery is now commonly preferred over open surgery [16,17,18]. In this case, given the severity of the patient’s POP and her overweight status, robot-assisted surgery was deemed more suitable. This approach provides superior 3D visualization and increased dexterity with wristed instruments, allowing for more precise dissection and suturing [19,20].

Fortunately, the patient recovered well without any additional complications. Follow-up visits at 1 month and 3 months post-surgery revealed no complications or recurrence. The patient is scheduled for outpatient visits at one-year intervals, and as of now, approximately 14 months postoperatively, there appear to be no complications or recurrence. Therefore, in high-risk patients with a high probability of developing or recurring POP, it may be advisable to perform the surgical correction of POP concurrently with hysterectomy.

This case is notable due to the cervical myoma, which, left untreated for an extended period, increased in size and resulted in uterine prolapse. Unlike similar case reports and publications where only a hysterectomy was performed, this case involved a concomitant SCP to minimize the potential for new POP, such as vault prolapse, following the hysterectomy [5,13,14]. Additionally, to ensure precision and minimize side effects, the Da Vinci Xi robot was utilized, resulting in favorable outcomes without recurrence over a follow-up period of approximately one year.

Given the patient’s limited access to medical care, which complicated postoperative management and lifestyle modifications (such as addressing constipation), this case underscores the effectiveness of performing concurrent POP correction surgeries in patients at high risk of recurrence. This approach distinguishes this report from others by highlighting the importance of combined surgical procedures to address POP in similar patient populations.

## 4. Conclusions

It is known that the risk of developing POP increases after a hysterectomy, regardless of the presence of POP prior to surgery. In patients with pre-existing POP, it is advisable to evaluate for high-risk factors of POP recurrence when planning a hysterectomy. In such cases, performing POP correction through SCP concurrently with the hysterectomy may be an effective treatment strategy.

## Data Availability

The datasets used during the current study are available from the corresponding author on reasonable request.

## Abbreviations

The following abbreviations are used in this manuscript:

SCP	Sacrocolpopexy;
POP	Pelvic organ prolapse;
APCT	Abdominal pelvis computed tomography ESBL Extended-spectrum beta-lactamase.
